# The validity and accuracy of 3D-printed patient-specific instruments for high tibial osteotomy: a cadaveric study

**DOI:** 10.1186/s13018-022-02956-2

**Published:** 2022-01-29

**Authors:** Zhuang Miao, Songlin Li, Desu Luo, Qunshan Lu, Peilai Liu

**Affiliations:** 1 Department of Orthopedics, Qilu Hospital, Cheeloo College of Medicine, Shandong University, Jinan, Shandong China; 2 Department of Orthopedic Surgery, Peking Union Medical College Hospital, Peking Union Medical College, Chinese Academy of Medical Science, Beijing, China; 3 Cheeloo College of Medicine, Shandong University, Jinan, Shandong China

**Keywords:** High tibial osteotomy, Patient-specific instrument, 3D-printed, Cadaveric study, Validity, Accuracy

## Abstract

**Objective:**

High tibial osteotomy (HTO) has been used for the treatment of patients with knee osteoarthritis. However, the successful implementation of HTO requires precise intraoperative positioning, which places greater requirements on the surgeon. In this study, we aimed to design a new kind of 3D-printed patient-specific instrument (PSI) for HTO, including a positioning device and an angle bracing spacer, and verify its effectiveness using cadaveric specimens.

**Methods:**

This study included ten fresh human lower-limb cadaveric specimens. Computed tomography (CT) and X-ray examinations were performed to make preoperative plans. PSI was designed and 3D-printed according to the preoperative plan. Then, the PSI was used to guide HTO. Finally, we performed X-ray and CT after the operation to verify its validity and accuracy.

**Results:**

The PSI using process was adjusted according to the pre-experimental procedure in 1 case. Hinge fracture occurred in 1 case. According to X-rays of the remaining eight cadaveric specimens, no statistically significant difference was noted between the preoperative planning medial proximal tibial angle (MPTA) and postoperative MPTA (*P* > 0.05) or the preoperative and postoperative posterior slope angle (PSA) (*P* > 0.05). According to the CT of 10 cadaveric specimens, no statistically significant difference was noted between the design angle and actual angle, which was measured according to the angle between the osteotomized line and the cross section (*P* > 0.05). The gap between the designed osteotomy line and the actual osteotomy line was 2.09 (0.8 ~ 3.44) mm in the coronal plane and 1.58 (0.7 ~ 2.85) mm in the sagittal plane.

**Conclusion:**

This 3D-printed PSI of HTO accurately achieves the angle and position of the preoperative plan without increasing the stripping area. However, its use still requires a certain degree of proficiency to avoid complications, such as hinge fracture.

## Introduction

HTO is primarily used in younger (< 60 years) patients with knee osteoarthritis caused by varus of the knee joint [1]. The principle of the procedure is to transfer the force line from the inflamed and worn medial compartment to the relatively normal lateral compartment through proximal tibial osteotomy, thereby relieving the symptoms of arthritis [2]. In the early days of HTO, it was thought to serve only to delay joint replacement [3, 4]. However, as the indications for HTO became clearer, the surgical technique improved, and the internal fixation advanced [5], its long-term outcome gradually increased with different investigators reporting 20-year survival rates of 85.1% [6].

The keys to HTO are the correct intraoperative bracing position and bracing angle. The surgical approach relies on the operator's experience and multiple intraoperative fluoroscopic views, and it is often difficult to achieve the preoperatively planned correction angle [2, 6]. PSI can be designed according to the patient's individual skeletal characteristics and preoperative plan. Through the fitting of the PSI to the patient's bone, the osteotomy position and bracing angle can be accurately marked to achieve the preoperative planned corrective angle and avoid the loss of bracing angle [7]. Its precise osteotomy features can also reduce the number of intraoperative fluoroscopic views [8] as well as the operation time [9]. The aim of this study was to design a PSI for HTO and apply this methodology to cadaveric bone to initially test its validity and accuracy.

## Materials and methods

### Specimens

Ten lower-limb specimens from the foot to 20 cm above the knee joint were obtained from five fresh human specimens. The inclusion criteria were as follows: (1) fresh lower extremity specimens from adult cadavers and (2) intact lower extremity specimens. Exclusion criteria included the following: (1) patients who previously underwent orthopedic surgery on the lower limbs and (2) patients with previous skeletal deformities of the lower limbs.

### Design of PSI

**Fig. 1:**
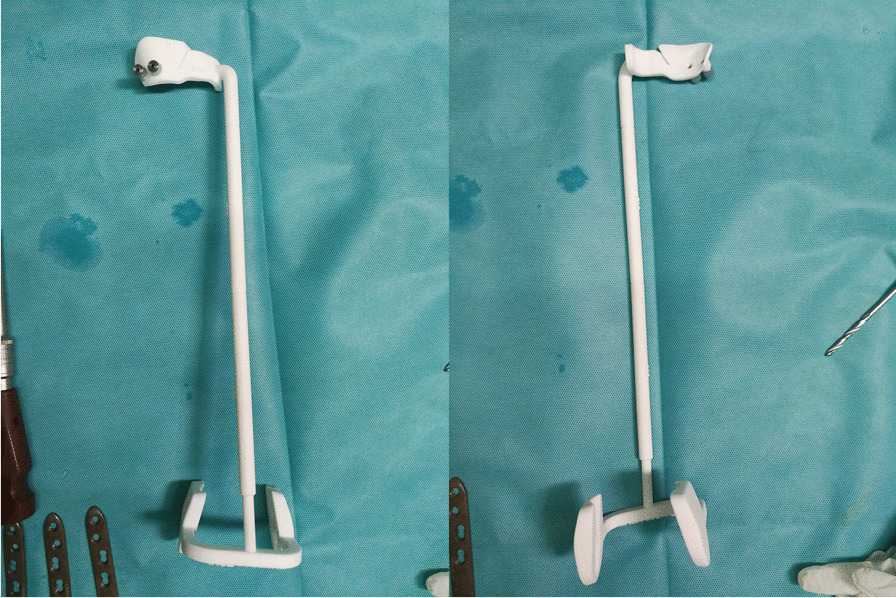
Positioning device (Left: outside view; Right: inside view; From upper to bottom: the proximal positioning device, the force line bar and the distal positioning device)

**Fig. 2:**
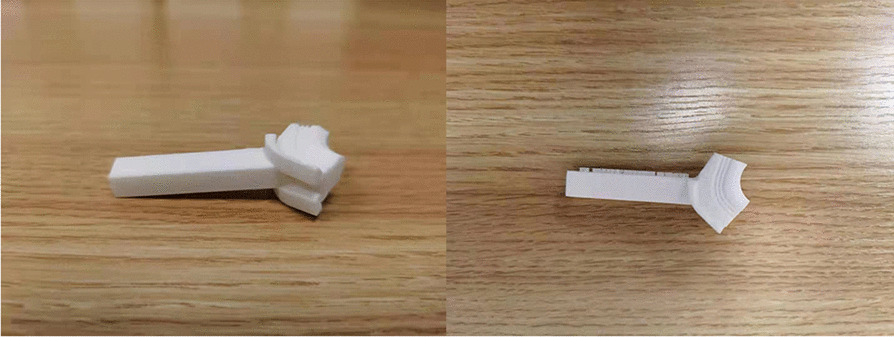
Angular bracing spacer (left: side view; right: top view)

The PSI consisted of a positioning device (Fig. 1) and an angular bracing spacer (Fig. 2). The positioning device includes three parts: the proximal positioning device, the distal positioning device, and the force line bar.

### Methods

**Fig. 3:**
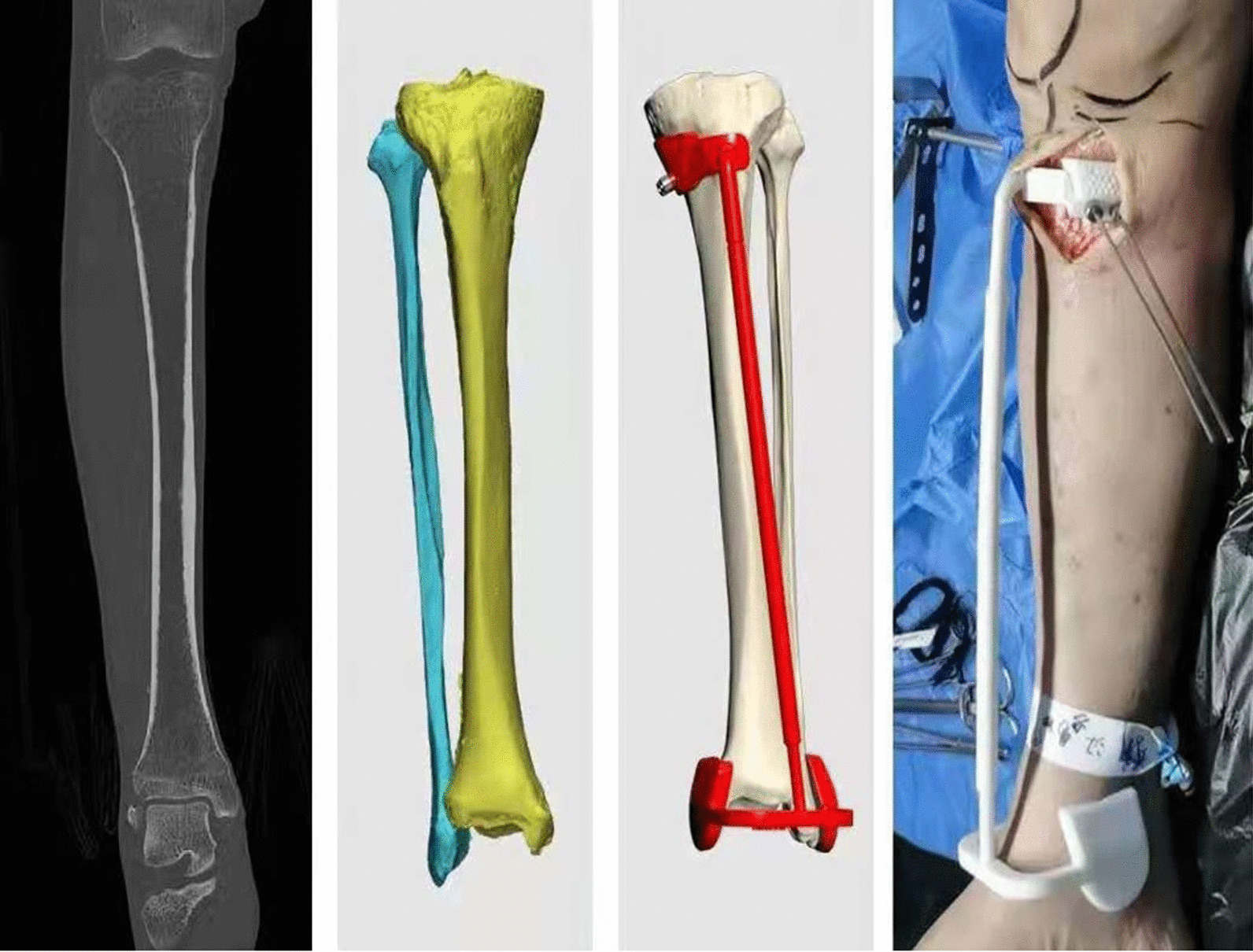
Design and 3D printing of PSI according to the preoperative plan and 3D skeletal model of the lower extremities (**a** perform CT examination. **b** CT scans were imported into Mimics 25.0 software to construct a 3D skeletal model of the lower limb. **c** The PSI was designed based on the preoperative plan and the 3D skeletal model of the lower extremities. **d** The PSI was 3D-printed)

**Fig. 4:**
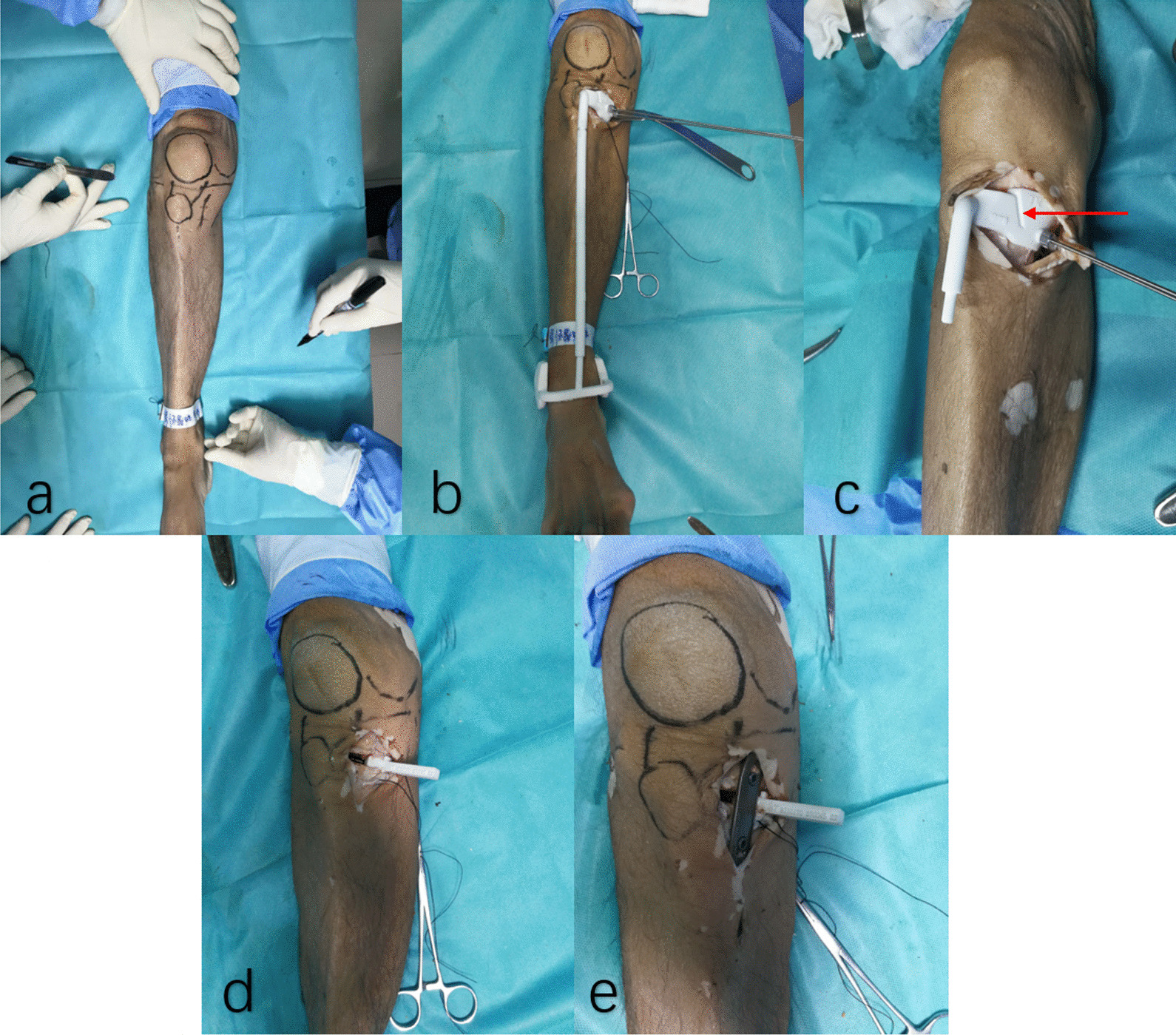
HTO using PSI on a cadaveric specimen (**a** Positioning body surface markers. **b** PSI was positioned according to body surface markers and two Kirschner wires were fixed. **c** A biplanar osteotomy was performed along the predetermined osteotomy channel (red arrow) in the PSI. **d** Insert angular bracing spacer. **e** Plate was installed and secured with eight screws)

**Fig. 5:**
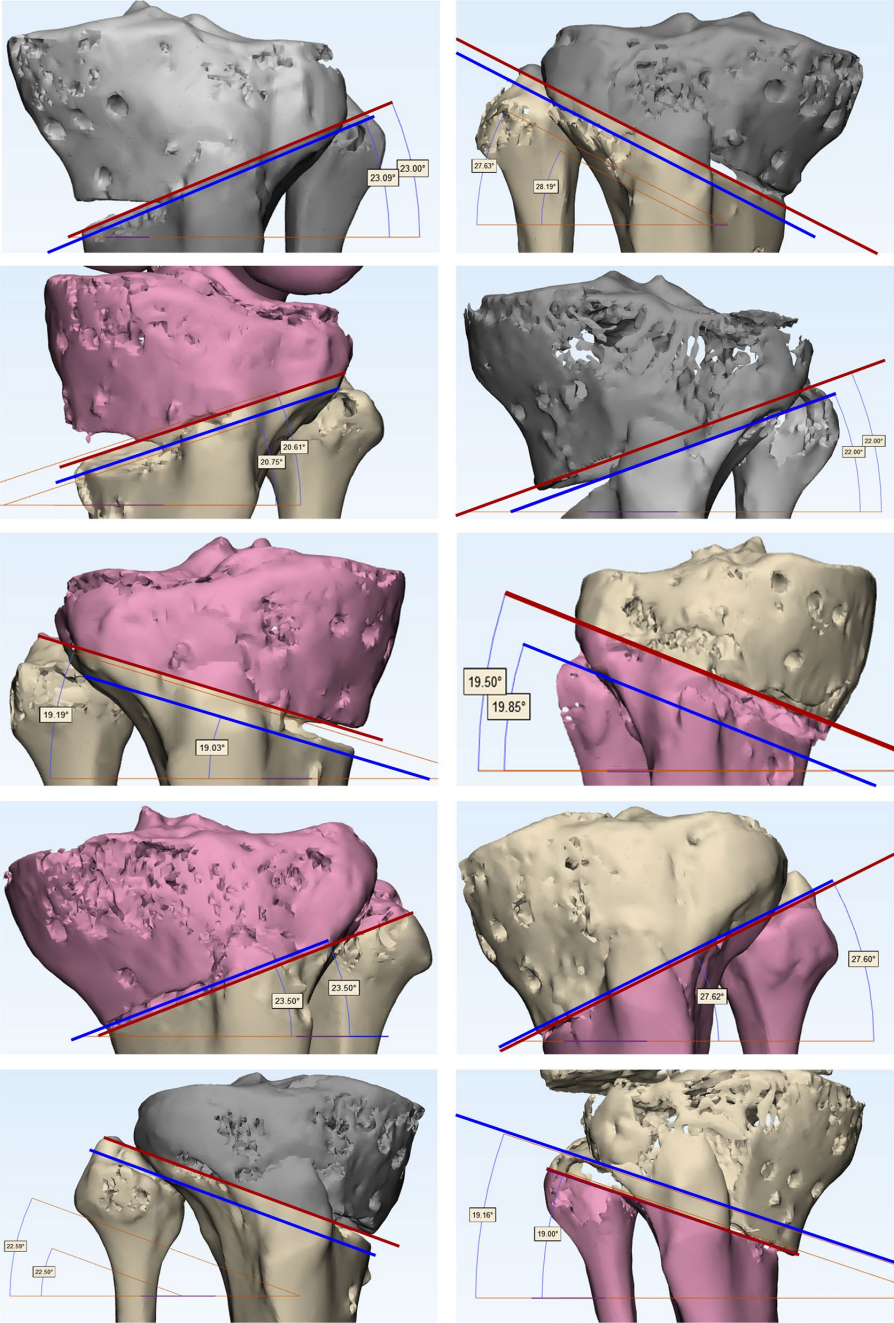
CT results of ten specimens (Red line: actual osteotomy line. Blue line: planning osteotomy line)

1. Lower-limb specimens from the foot to 20 cm above the knee joint were obtained from fresh human specimens, and CT thin-layer scans (layer thickness 1 mm) and X-ray examinations were performed.
2. DICOM 3.0 format data from CT scans were imported into Mimics 25.0 software to construct a 3D skeletal model of the lower limb.
3. X-rays were obtained in orthogonal and lateral views, and two observers who were not involved in the surgery and were unaware of the surgical plan measured the MPTA and PSA.
4. Preoperative planning was performed based on the radiographs to determine the postoperative target MPTA. The PSI was designed and 3D-printed based on the preoperative plan and the 3D skeletal model of the lower extremities (Fig. 3).
5. HTO was performed on the specimen using the PSI. The surgical procedure was as follows: a 5-cm-long surgical incision was made medial to the patellar ligament, and the skin and subcutaneous tissue were incised sequentially to expose the superficial layer of the medial collateral ligament and subperiosteal dissection. A positioning device is installed, and two Kirschner wires are inserted along the preset channel of the PSI to fix the proximal positioning device after both the proximal and distal ends are affixed. A biplanar osteotomy was performed along the predetermined osteotomy channel in the PSI. The angular bracing spacer was inserted after the osteotomy surface was opened with a splitter. The angular bracing spacer is considered to achieve the target spacer angle after being fully seated in the depth-limiting groove. The proximal medial tibial plate was installed and secured with eight screws. Finally, the angular bracing spacer was removed, and the wound was sutured (Fig. 4).
6. Postoperative X-rays were obtained. The same two observers measured the postoperative MPTA and PSA.
7. CT was performed after plate removal to construct a postoperative 3D skeletal model of the lower extremity, which was compared with the preoperative plan and analyzed for gaps in the osteotomy line angle and osteotomy line position (Fig. 5).

### Statistical analysis

Data analysis was performed using SPSS 23.0 software. The intragroup correlation coefficient (ICC) was used to assess interobserver reliability by rating the degree of agreement of measurements across observers. ICC ≥ 0.75 indicates very good reliability, 0.60 ≤ ICC < 0.74 indicates good reliability, 0.40 ≤ ICC < 0.60 indicates credible, and ICC < 0.40 indicates poor reliability. Paired t tests were used to rate preoperative planning versus postoperative MPTA, PSA and angle of osteotomy line. Here, *P* < 0.05 indicates a significant difference.

## Results

The angular bracing spacer was removed before the plate was fixed in the pre-experimental test (Specimen No. 10), resulting in a large difference between the angle after fixation and the design angle. Subsequent modifications were made to the angular bracing device and the operating procedure, so the data were not included in the analysis. Hinge fracture occurred in Specimen No. 6. X-ray suggested a Takeuchi III fracture, and angular data could not be measured. Thus, this specimen was not included in the analysis. A total of eight X-ray specimens and ten CT specimens were analyzed.

### Degree of agreement in observer measurements

ICC was 0.909 in MPTA and 0.400 in PSA. The results show that the reliability of the MPTA observation is very good, and the reliability of the PSA observation is credible. Considering the existence of multiple methods for the measurement of PSA [10], as long as the preoperative and postoperative measurement methods of the same observer during the PSA determination are consistent, the slightly lower confidence of the PSA observation results does not affect the determination of the experimental results.

### MPTA

The preoperative MPTA was 86.22°(82 ~ 90°), and the spacer angle was 9.50° (7 ~ 13°). The preoperative planned MPTA was 95.72° (94 ~ 98°) and the postoperative MPTA was 95.00° (93 ~ 99°). The difference between the postoperative MPTA and the preoperative planned value was − 0.72° (− 3 ~ 2°). The difference between the MPTA preoperative planned versus actual postoperative values was not statically significant (*P* = 0.083).

### PSA

Observer I reported a pre- and postoperative PSA of 9.50° (6 ~ 14°) and 9.13° (8 ~ 12°), respectively, with a preoperative to postoperative difference of − 0.38° (− 4 ~ 4°). Observer II reported a pre- and postoperative PSA of 8.06° (7 ~ 11°) and 7.25° (6 ~ 10°), respectively, with a preoperative-to-postoperative difference of − 0.81° (− 3 ~ 2.5°). The difference between the PSA preoperative versus postoperative inclination angle was not statically significant (*P* = 0.310).

### The gap and angle difference of osteotomy line

The angle between the osteotomy line and the cross section measured by CT was 22.56° (19.03 ~ 28.19°). The actual angle was 22.46° (19 ~ 27.63°), and the relative angle difference was − 0.10° (− 0.56 ~ 0.16°). The difference between the preoperative planned angle of osteotomy line and the cross section versus actual postoperative values was not statically significant (*P* = 0.165).The gap between the designed osteotomy line and the actual osteotomy line was 2.09 (0.8 ~ 3.44) mm in the coronal plane and 1.58 (0.7 ~ 2.85) mm in the sagittal plane.

## Discussion

The current design challenges of 3D-printed PSI mainly involve how to achieve the precise osteotomy position and how to ensure the osteotomy angle. The most commonly used method to achieve precise osteotomy position involves the use of bony landmarks in the vicinity of the osteotomy position, so the osteotomy position can be precisely determined by fitting the PSI to the bony landmarks near the osteotomy [7, 11]. However, the anatomical pattern of the proximal tibia is relatively flat, and specific anatomical landmarks are not easily found. Additionally, the exposure of bony markers causes an enlargement of the intraoperative incision, an increase in soft tissue stripping, and a relative increase in trauma. In recent years, some studies have proposed using distal bony markers to precisely position the osteotomy [12], solving the problem of increased intraoperative exposure. However, the degree of accuracy is reduced compared with that of proximal bony markers. Numerous operation methods are available to ensure the bracing angle. Some studies add calibration holes at the proximal and distal ends of the PSI, and the calibration holes can only be calibrated when the bracing angle reaches the target value [7]. The PSI designed by Victor et al. is positioned by proximal bony markers and can be punched through the PSI. The peg tract on bone can be aligned with the peg tract of the internal fixation after the bracing angle is reached, but the prepunched peg tract can impact the intraoperative plate adjustment [8].

In this study, we designed a PSI for HTO. In terms of precise positioning, the PSI was designed with reference to both the proximal anatomic landmarks of the tibia and the distal landmarks of the inner and outer ankle with a greater three-dimensional positioning span. The inner surface of the proximal positioning device fits the bone surface near the osteotomy line. There are two grooves on the inner side of the distal positioning device, which were 3D-printed according to CT to match the inner and outer ankle bulges. The use of both proximal and distal positionings solves the disadvantage of a large stripping area when using proximal positioning alone and provides more accurate positioning without increasing the proximal exposure. Additionally, the proximal positioning device is connected to the distal positioning device using a force line bar, allowing for an initial assessment of the extent of force line correction. In terms of precise force line correction, the design of the PSI uses an angle bracing spacer to achieve the preoperative design of force line correction and to determine the placement of the plate while avoiding the loss of spacer angle during the plate locking process.

The ability to correct the MPTA reflects the degree of accuracy of the PSI for force line correction. When comparing the postoperative MPTA with the planned MPTA, no significant difference was found, indicating that the degree of force line correction by the PSI was accurate and could achieve the preoperative planned degree of force line correction in the absence of errors in measurement and operation. Different previous studies reported the application of PSI for HTO with a mean difference between the preoperative planned and postoperative MPTA of approximately 0.1° ~ 1.9° [13, 14]. The mean difference between the postoperative MPTA and preoperative planned value in this study was 0.72°, which was similar to the results reported in the previous literature.

The PSA represents the angle of inclination of the tibial plateau. Previous studies have generally concluded that closed-wedge osteotomy decreases the tibial plateau PSA, whereas open-wedge osteotomy increases the tibial plateau PSA [15, 16]. In addition, an increase in postoperative PSA accelerates anterior cruciate ligament degeneration [17], leading to knee instability and accelerating postoperative knee degeneration. The current study concluded that the change in the PSA after medial open-wedge HTO is closely related to the orientation of the hinge axis during the osteotomy [18]. Wang et al. proposed that the posterior tibial cortex should be completely cut off during the osteotomy, and only the lateral cortex should be retained as the hinge axis [19]. The lateral cortex points in the anterior–posterior direction and corrects the varus deformity only in the coronal plane and does not cause any change in the PSA. In this study, the direction of the osteotomy line and the osteotomy depth were guided by the predefined osteotomy slot in the PSI, which could effectively avoid the change in the PSA caused by the osteotomy.

Hinge fracture is a common complication of HTO that often occurs during wedge gap sparing with an incidence of approximately 19.8% ~ 30.4% as previously reported in the literature [20, 21]. Takeuchi classified hinge fractures into three categories based on the relative position of the fracture to the upper tibiofibular joint [22] with type I being stable and types II and III being unstable. Hinge fractures may lead to delayed healing at the osteotomy and loss of corrective angulation and affect patient satisfaction postoperatively [23]. In a systematic review of 11 studies, Kim et al. found that the occurrence of hinge fractures was associated with greater wedge gap spreading with spreading gaps of approximately 11.40 ~ 12.60 mm in the hinge fracture group and 9.80 ~ 11.12 mm in the nonhinge fracture group [24]. One hinge fracture occurred in this study. After postoperative X-ray and CT scan analysis, its occurrence was related to the large brace gap, and the osteotomy depth did not reach the preoperative plan. The results demonstrate that the use of PSI reduces the occurrence of complications, such as hinge fracture, but its use still requires a certain degree of proficiency.

The following limitations of this study should be noted: (1) Only 10 cadaveric specimens were included in this study, which is a small number of specimens. (2) This study used lower extremity cadaveric bone specimens for testing. Although these specimens were able to simulate bony structures, repeated freezing and thawing of cadaveric bone specimens could not simulate the effects of soft tissue on intraoperative positioning and surgical manipulation. (3) The specimens used in this study were not lower extremity specimens with knee osteoarthritis and varus deformity, and differences between the set bracing angle and the actual surgical target bracing angle were noted. Further testing is needed to observe whether the same precise results can be achieved with clinical surgical application.

## Conclusion

In conclusion, the PSIs for HTO developed in this study can precisely achieve the degree of force line correction designed preoperatively, osteotomy position and bracing angle without increasing the stripping area while avoiding the change of PSA after osteotomy. However, its use still requires a certain degree of proficiency to avoid complications, such as hinge fractures.

## Data Availability

The data used and/or analyzed during the current study are available from the corresponding author on reasonable request.

## Abbreviations

PSI: Patient-specific instrument
HTO: High tibial osteotomy
CT: Computed tomography
MPTA: Medial proximal tibial angle
PSA: Posterior slope angle
